# Spontaneous Intracranial Hypotension Without CSF Leakage—Concept of a Pathological Cranial to Spinal Fluid Shift

**DOI:** 10.3389/fneur.2021.760081

**Published:** 2021-11-01

**Authors:** Johannes Goldberg, Levin Häni, Christopher Marvin Jesse, Irena Zubak, Eike I. Piechowiak, Jan Gralla, Tomas Dobrocky, Jürgen Beck, Andreas Raabe

**Affiliations:** ^1^Department of Neurosurgery, Inselspital, Bern University Hospital, University of Bern, Bern, Switzerland; ^2^University Institute of Diagnostic and Interventional Neuroradiology, Inselspital, Bern University Hospital, University of Bern, Bern, Switzerland; ^3^Department of Neurosurgery, Freiburg University Hospital, Freiburg, Germany

**Keywords:** spontaneous intracranial hypotension, compliance, CSF physiology and anatomy, CSF leak or fistula, orthostatic headaches

## Abstract

**Objective:** Spontaneous intracranial hypotension (SIH) is typically caused by CSF leakage from a spinal dural tear, a meningeal diverticulum, or a CSF venous fistula. However, some patients present with classic orthostatic symptoms and typical intracranial imaging findings without evidence of CSF leakage despite repeated diagnostic work-up. This article aims to elaborate a hypothesis that would explain a pathologically increased orthostatic shift of CSF from the cranial to the spinal compartment in the absence of a CSF leak.

**Medical Hypothesis:** The symptoms of SIH are caused by a decrease in intracranial CSF volume, intracranial hypotension, and downward displacement of intracranial structures. A combination of pathologically increased spinal compliance, decreased intracranial CSF volume, low CSF outflow resistance, and decreased venous pressure might result in a pathological orthostatic cranial-to-spinal CSF shift. Thus, in rare cases, intracranial hypotension may occur in the absence of CSF leakage from the dural sac.

**Conclusion:** We propose a pathophysiological concept for the subgroup of SIH patients with typical cranial imaging findings and no evidence of CSF leakage. In these patients, reducing the compliance or the volume of the spinal compartment seems to be the appropriate therapeutic strategy.

## Introduction

**Figure 1:**
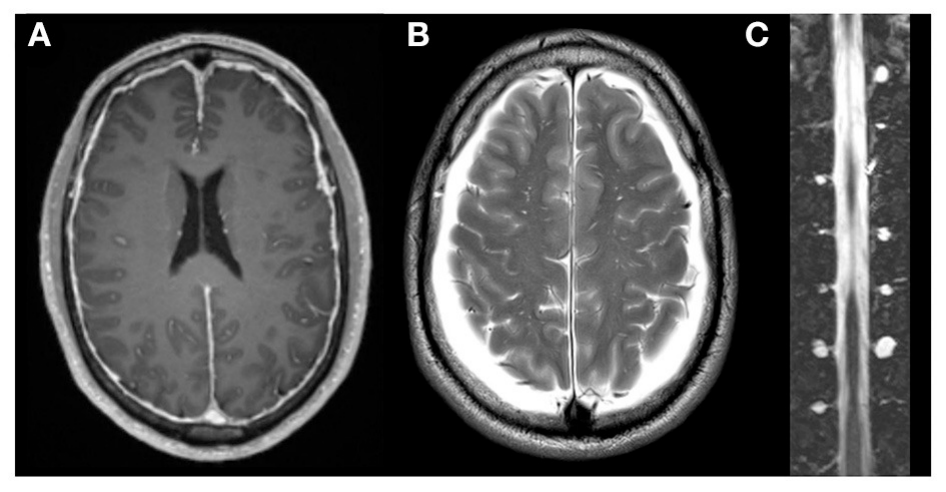
Imaging of a patient with SIH symptoms without evidence of CSF leakage or CSF venous fistula. A 68-year-old male patient exhibiting classic SIH symptoms with typical cranial imaging findings, such as dural enhancement **(A)** and bilateral subdural hygroma **(B)** and no evidence of CSF leakage or CSF venous fistula despite repeated diagnostic work-up. Spinal MRI revealed a large number of prominent meningeal diverticula **(C)**.

Orthostatic headache is the hallmark of headache attributed to spontaneous intracranial hypotension (SIH) as defined by the third edition of the international classification of headache disorders (ICHD-3) category 7.2.3 (1). The effects of the disease can be devastating and cause disability. Leakage of CSF from a defect in the spinal dura mater is responsible for a pathological decrease of CSF volume (2–4). Intracranial CSF volume depletion when in an upright position leads to a pathological decrease of intracranial pressure (ICP), brain sagging, venous distension (5), and related symptoms such as orthostatic headache and cranial nerve deficits (2, 6). In patients with persistent SIH symptoms, invasive diagnostics such as dynamic myelography and CT-myelography are recommended to search for spinal CSF leaks that are amenable to surgical treatment like ventral dural tears, ruptured meningeal diverticula, or a direct CSF to venous fistula (4, 7–11). However, in some patients with intractable SIH symptoms and typical cranial imaging findings (12), no evidence of spinal CSF leakage can be found despite repeated diagnostic work-up (Figure 1). In our experience, this subgroup accounts for up to 10–15% of patients, which is in line with the observations of Schievink et al. (13). Apart from symptomatic treatment, appropriate further management of these patients is unclear.

Orthostatic headache without CSF leakage due to a pathologically increased spinal compliance was first discussed by Hunderfund and Mokri (14). However, it is not yet fully understood and is rarely considered in clinical practice. In this paper, we sought to corroborate the hypothesis of a pathological cranial-to-spinal CSF shift without CSF leakage, to consider the factors that may contribute, and to discuss the diagnostic and therapeutic options.

**Figure 2:**
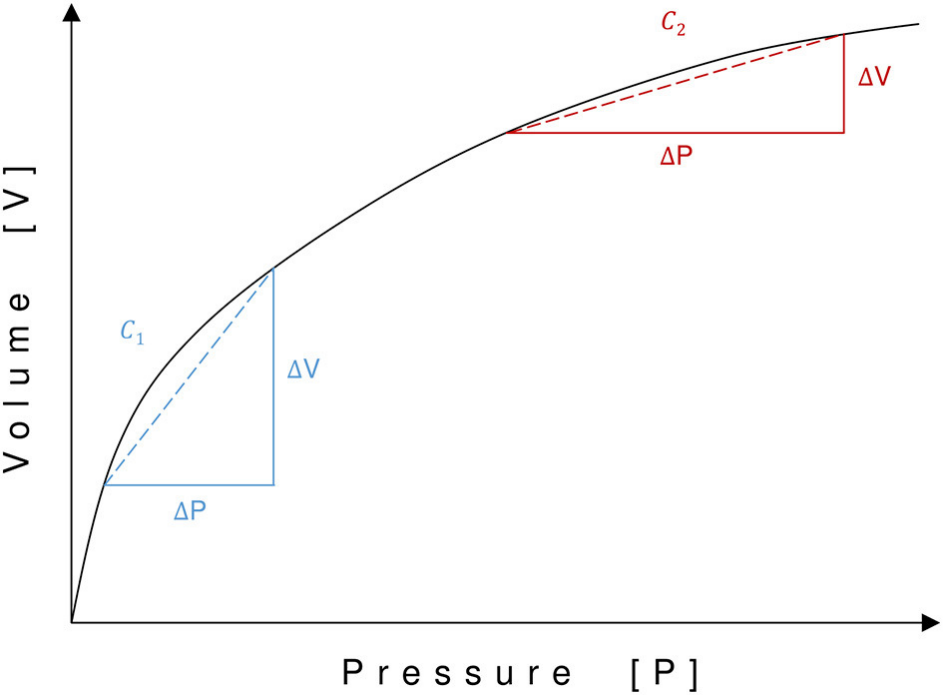
Pressure-volume curve of the craniospinal compartment. Pressure (P) is displayed on the x-axis and volume (V) on the y-axis. Compliance (*C*) is represented by the slope of the pressure–volume curve. Due to its exponential function, compliance varies with different pressure–volume status. A shift to the left on the pressure–volume curve leads to increased compliance (*C*1), whereas a shift to the right leads to decreased compliance (*C*2).

**Figure 3:**
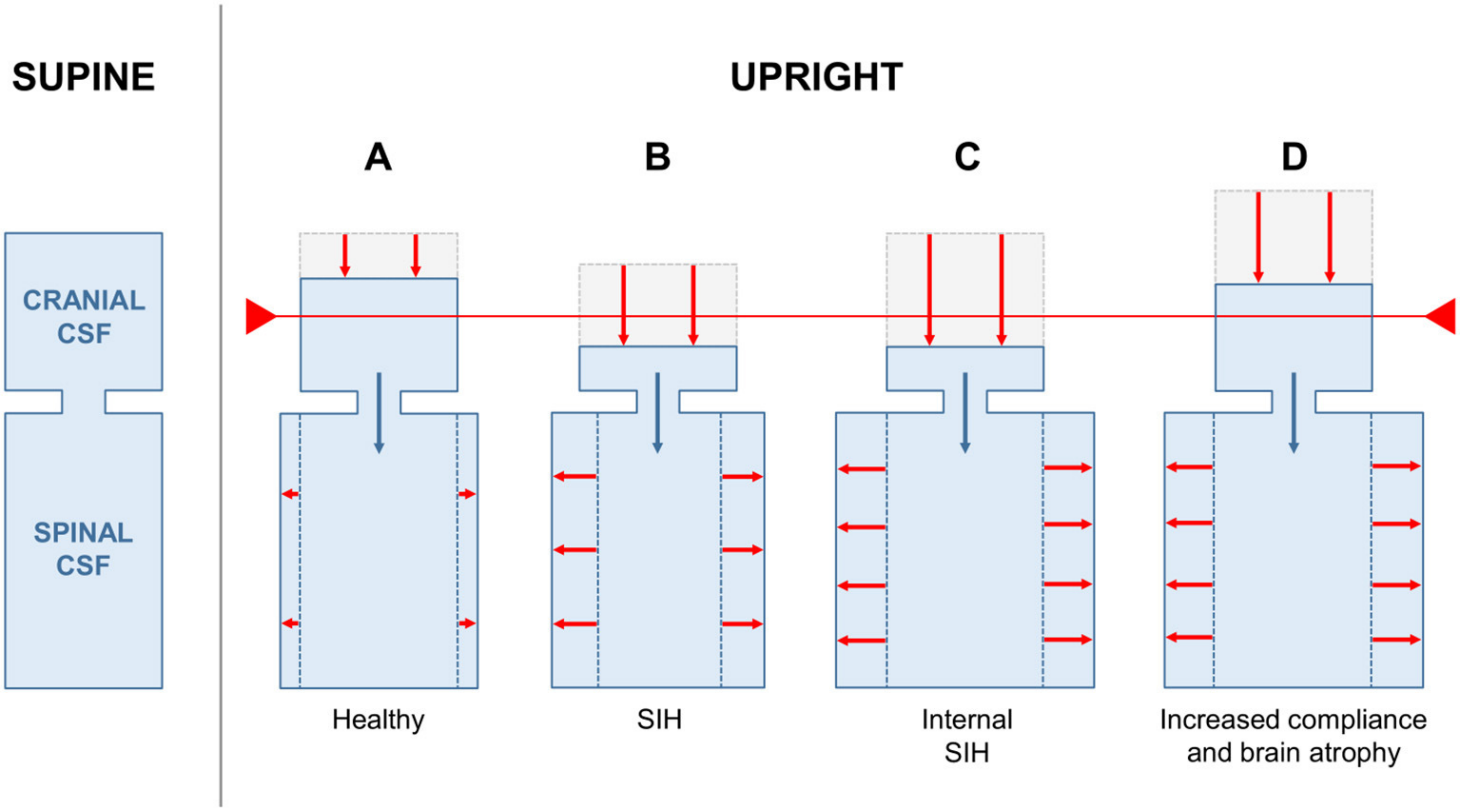
Hypothetical CSF volume shifts in different constellations of SIH and healthy individuals. Schematic illustration of craniospinal CSF distribution in the supine (left) and upright position (right; **A–D**). The red line indicates the threshold for development of orthostatic symptoms. In a healthy individual only a small proportion of the cranial CSF volume is shifted to the spinal compartment after a positional change **(A)**. CSF leakage in patients with classical SIH leads to CSF hypovolemia and a shift to the left on the pressure–volume curve with increased compliance. Thus, a larger volume (absolute and proportional) is shifted to the spinal compartment **(B)**. In a state of increased spinal compliance, a larger absolute volume is shifted to the spinal compartment **(C,D)**. In patients with normal or low intracranial CSF volume, the proportion of CSF shifted is large and causes internal SIH **(C)**. In patients with high intracranial CSF volume (e.g., because of brain atrophy) the proportionally smaller CSF shift is compensated and no symptoms occur **(D)**.

**Figure 4:**
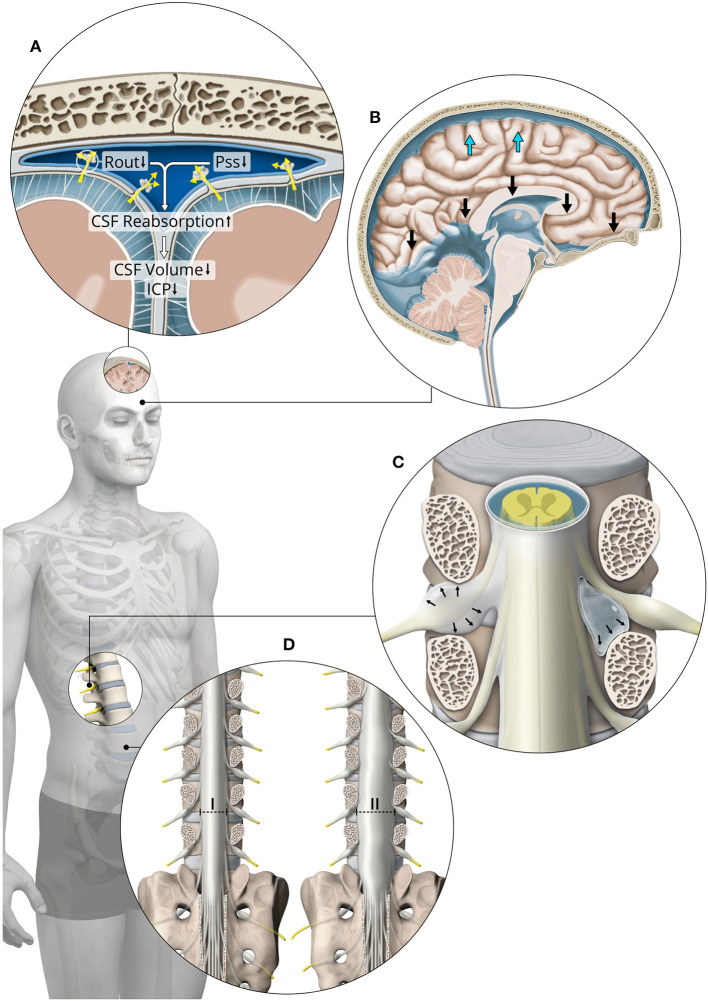
Overview of factors contributing to internal SIH. Predisposing factors are **(A)** low CSF outflow resistance (Rout), low intracranial venous pressure (Pss) and **(B)** low intracranial CSF volume. The main pathophysiological factor is increased spinal compliance due to **(C)** meningeal diverticula, dural tears with prolapsing arachnoid around meningeal diverticula or **(D)** increased distensibility of the dura mater (I = normal distensibility, II = increased distensibility). A combination of these factors can lead to a pathological cranial-to-spinal CSF shift causing brain sagging **(B)** and orthostatic symptoms without CSF leakage.

Figures 2–**4** are displaying the physiological and pathophysiological concepts of CSF dynamics relevant for SIH that are going to be elaborated in detail in the following.

## Physiology of Craniospinal CSF Dynamics Relevant for SIH

### Physiological Intracranial and Intraspinal Pressure Dynamics During Positional Changes

In a person in a recumbent position, the CSF pressure is equal along the craniospinal axis. Thus, the opening pressure during lumbar puncture in a recumbent position corresponds to the ICP. When moving to an upright position, the pressure redistributes along the hydrostatic column from negative values in the cranial compartment to positive values in the spinal compartment (15). The zero pressure point, i.e., the transition point from negative to positive pressure values, is located in the upper cervical spine (15). The point where the pressure in the upright position is identical to the pressure in the recumbent position is called the hydrostatic indifferent point (HIP). It is physiologically situated between C7 and T5 (15). The physiological ICP in the upright position is slightly negative (16–18).

### Craniospinal Compliance

Craniospinal compliance (*C*) is a measure of distensibility. It describes the ratio between changes in volume and pressure $\left(\;C\;=\;\frac{\Delta V}{\Delta P}\right)$ and corresponds to the slope of the pressure–volume curve. Since the relationship between pressure and volume in the CSF space is exponential, compliance increases with a lower filling pressure or a lower CSF volume and vice versa (19, 20). Thus, in a state of high baseline pressure, a change in volume results in a relatively larger change of pressure than in a state of low baseline pressure (Figure 2).

It is essential to understand the normal movements of CSF between the cranial and the spinal compartment caused by postural changes. The craniospinal CSF system of adults accommodates ~150 (125–200) ml (21). Roughly two-thirds of the CSF volume is located in the spinal and one-third in the cranial compartment. When moving from a recumbent to an upright position, the pressure above the HIP decreases and the pressure below the HIP increases. Because of the compliance of the thecal sac, the spinal compartment accommodates some additional CSF volume, causing a CSF shift from the cranial to the spinal compartment, and a decrease in ICP. Provided that the spinal compliance is physiological, the cranial-to-spinal CSF shift is limited to a few milliliters of CSF and the decrease in ICP remains within a physiological range (22), which is not associated with orthostatic headache (Figure 3A).

## Pathophysiology of CSF Dynamics in SIH

### Pathophysiology of Intracranial Pressure Changes in SIH Patients With Proven CSF Leakage

In patients with CSF leakage, the total craniospinal CSF volume is reduced, and the pressure decreased (23). Therefore, the state of the craniospinal compartment shifts to the left on the pressure–volume curve with increased compliance (Figure 2). When a patient with CSF leakage moves from a recumbent to an upright position, because of its increased compliance, the spinal compartment can accommodate more CSF. Thus, a larger proportion of the already diminished intracranial CSF volume is now shifted into the spinal compartment, and a pathologically low ICP, brain sagging, and orthostatic headache and other symptoms (Figure 3B) can occur. In patients who experience orthostatic headache after lumbar puncture or due to over-draining CSF shunts, such a pathologically low ICP can decrease to −35 cmH_2_O in an upright position (16, 17, 24).

Due to the equal distribution of pressure and fluid when in a horizontal position, the reduced CSF volume and the increased compliance have only a minor effect on ICP in the recumbent position. Measurements of opening lumbar pressure of SIH patients in the recumbent position demonstrate lower (i.e., <6 cmH_2_O) or most often normal but not negative pressure. Thus, in the recumbent position, the intracranial pressure is positive, even in patients with CSF loss, which prevents brain sagging and explains the immediate relief experienced when the patient is lying down.

## The Hypothesis of Internal SIH—Factors Contributing to Intracranial Hypotension Without CSF Leakage

We hypothesize that a pathologically increased compliance of the spinal compartment with increased orthostatic CSF shift from the cranial to the spinal compartment can cause intracranial hypotension and brain sagging in the absence of CSF leakage. Together with predisposing factors, such as low total and low intracranial CSF volume, decreased CSF outflow resistance, and decreased intracranial venous pressure, orthostatic symptoms can occur. We believe that this concept applies to patients exhibiting classic SIH symptoms with typical intracranial imaging findings, but no evidence of CSF leakage despite repeated diagnostic work-up. We will refer to this as *internal SIH* in the following sections.

### Increased Compliance of the Spinal Compartment

The volume of CSF shifting from the cranial toward the spinal compartment during a postural change depends mostly on the compliance and thus on the volume reserve of the spinal compartment, the latter being the main contributor to the total craniospinal compliance (25). In an *a priori* pathological increase in spinal compliance, the volume of CSF shifting from the cranial to the spinal compartment may be large enough to cause SIH, brain sagging, and symptoms, without CSF leakage to the extradural space (Figure 3C). We consider this the major pathophysiological factor for internal SIH.

Potential explanations for pathologically increased compliance are (1) a large number of meningeal diverticula (Figure 1C), (2) dural tears with prolapsing arachnoid around meningeal diverticula or elsewhere (Figure 4C), and (3) increased distensibility of the dura mater (Figure 4D) (4, 26, 27). According to Schievink, meningeal diverticula of 8 mm or more are accountable for 42% of cases of SIH. However, an associated extradural CSF collection was found in only 22% of these cases (13). A tear in the dura mater may lead to a prolapse of the arachnoid, which is frequently found intra-operatively when exploring meningeal diverticula. The prolapsing arachnoid could potentially increase the compliance of the spinal CSF compartment. To our knowledge, imaging of those diverticula has only been done in the supine position, and it is not known whether ballooning of diverticula when in the upright position might increase the compliance of the spinal compartment. Additionally, Kranz et al. found significantly more meningeal diverticula in SIH patients without evidence of CSF leakage than in SIH patients with a proven CSF leak (28). Therefore, we hypothesize that meningeal diverticula can result in increased compliance of the spinal compartment, leading to SIH symptoms, despite the absence of CSF leakage.

### Low Total CSF Volume

In the case of an overall reduction in CSF volume, the state of the craniospinal compartment shifts to the left on the pressure-volume curve, leading to increased compliance (Figure 2). This might allow for a pathologically increased shift of CSF volume to the spinal compartment when moving to an upright position.

### Low Intracranial CSF Volume

The intracranial CSF volume varies according to the size of the ventricles and the volume of the subarachnoid space. It is age-dependent but also related to a variety of factors causing brain atrophy. If the brain is atrophic, the size of the intracranial CSF compartment is increased. For example, provided that the same amount of volume in milliliters shifts to the spinal compartment during positional changes, in patients with *higher* intracranial CSF volume the proportion of CSF shifted is *lower* and more CSF remains in the intracranial space (Figure 3D). Conversely, in patients with *lower* intracranial CSF volume, the proportion of CSF shifted is *higher* and less CSF remains in the intracranial space (Figure 3C). A smaller intracranial CSF volume predisposes an individual to a reduced buoyancy of the brain, brain sagging and increased tension on pain sensitive structures like meninges and dural sinuses (Figure 4B). The contribution of smaller intracranial CSF volume is supported by the age distribution of SIH patients who are seldom elderly.

### CSF Outflow Resistance

ICP in the recumbent position is a function of CSF formation rate (*I*_*f*_), CSF outflow resistance (*R*_out_), and sagittal sinus pressure (*P*_ss_), as defined in Davson's equation: (17).


$$\begin{array}{l} ICP=I_{f}*R_{out}+P_{ss} \end{array}$$

The outflow of CSF is directly proportional to the pressure difference between ICP and *P*_ss_. The proportionality constant is *R*_out_ (29, 30). *R*_out_ can be determined by continuous lumbar infusion testing (31). Decreased *R*_out_ leads to lower CSF volume and pressure due to increased CSF reabsorption (Figure 4A). Therefore, the state of the craniospinal compartment shifts to the left on the pressure–volume curve with increased compliance.

A decrease of *R*_out_ might occur in the form of a spinal CSF venous fistula. Although rare it is a recognized cause for the development of SIH (13, 32, 33). Another hypothesis of unknown clinical relevance is a pathologically decreased *R*_out_ caused by high resorption capacity of the spinal meningeal roots. Although they play a role in CSF reabsorption, their normally constant and unchanged appearance does not sufficiently explain the sudden onset of SIH symptoms. However, multiple meningeal diverticula around spinal nerve roots might form throughout life and are a potential cause of an acquired increased CSF reabsorption capacity and sudden onset of SIH symptoms. However, this is speculative and needs to be proven.

### Decreased Venous Pressure

If *R*_out_ remains constant, according to the Davson's equation a decrease in intracranial or intraspinal venous pressure will lead to increased CSF reabsorption, reduced CSF volume, lower ICP, and a shift toward the left on the pressure–volume curve with increased compliance (Figure 4A). Moreover, a reduction of spinal epidural venous filling allows the thecal sac to expand more easily in case of volume load. Kumar and colleagues reported a case of intracranial hypotension associated with inferior vena cava stenosis (34). Low intracaval pressure has been proposed to cause increased CSF reabsorption from the spinal compartment to the inferior vena cava via the epidural venous plexus (34, 35). Symptoms and imaging findings resolved after stenting of the stenotic segment.

## Discussion

We describe the hypotheses of a pathological, orthostatic cranial-to-spinal CSF shift resulting in the clinical picture of symptomatic intracranial hypotension without evidence of CSF leakage, which we refer to as *internal SIH*. Based upon the interaction of pathologically increased compliance of the spinal compartment with predisposing factors such as decreased total and intracranial CSF volume, decreased CSF outflow resistance, and decreased venous pressure, intracranial hypotension and brain sagging may develop. This hypothesis is important because the contributing pathophysiological factors could ultimately serve as diagnostic and therapeutic targets.

### Criteria Qualifying for the Suspicion of Internal SIH

We propose the following combination of criteria for the suspicion of internal SIH:

1. *Classic SIH symptoms*
2. *Typical cranial imaging findings* *(**12**)*
3. *Absence of evidence of CSF leakage or CSF venous fistula despite repeated spinal imaging after intrathecal contrast application (MR-myelography, CT-myelography, dynamic digital subtraction myelography)* *(**4**)*
4. *Clear benefit from unspecific epidural blood patch (EBP), upon repeated applications*.

These criteria do not provide direct proof and thus allow only the suspicion of internal SIH. Several problems need to be considered when trying to establish this diagnosis: First, because of the limited sensitivity of MR-myelography, CT-myelography, dynamic CT-myelography, and digital subtraction myelography, it is not possible to exclude an existing, but undetected small dural leak or CSF venous fistula. Thus, repeated and targeted work-up is required before considering the diagnosis of internal SIH.

Second, the above-mentioned factors—spinal compliance, total CSF volume, intracranial CSF volume, CSF outflow resistance, and low venous pressure—are relevant to the hypothesis of internal SIH. However, routine testing of these factors is not yet accepted in clinical practice. Furthermore, no specific test with diagnostic thresholds exists for these parameters. For instance, even the most promising theoretical parameter, R_out_, shows considerable variations and time-dependent behavior (31).

Direct proof could be obtained from measurement of pathologically increased compliance by using the opening pressure and the volume-pressure response, and by ICP measurement while moving into an upright position. Indeed, some patients with severe intractable symptoms would qualify for invasive and dynamic ICP measurement with an implanted telemetric ICP probe. However, the insertion of these devices exposes the patient to a relevant risk of neurological deficits (0.4%), seizures (4.5%), and infections (1.6%) (36). Hence, physicians have been reluctant to use them as a diagnostic tool in headache attributed to spontaneous intracranial hypotension.

From a pathophysiological point of view, we also advocate measurement of R_out_ by lumbar infusion testing, and CSF volumetry of cranial and spinal spaces in the diagnostic work-up of these patients.

### Therapeutic Options

Of all the contributing factors described above, addressing the pathologically increased spinal compliance and thus the spinal volume reservoir is most feasible. A simple and well-tolerated method to achieve this is EBP (37, 38). The temporary relief of SIH symptoms after unspecific EBP is in line with the hypothesis of internal SIH. Increasing the volume of the epidural space with an EBP leads to compression of the dural sac, reduction of the volume capacity and decreased compliance of the spinal CSF compartment. Thus, the volume of CSF that shifts from the cranial to the spinal compartment during a postural change is smaller and more CSF remains in the intracranial space. The temporary nature of the relief is most likely explained by the absorption of blood from the epidural space over time and the restoration of the increased compliance. For patients with a good but temporary clinical response, repeated EBP is an option. In patients with a sustained response to EBP, the blood might have initiated a process of scarring in the epidural space and/or the spinal dura mater, thereby diminishing the compliance of the spinal compartment, or the site of leakage could have closed spontaneously.

Another therapeutic option to reduce compliance of the spinal compartment is reduction of prominent meningeal diverticula or dural reduction surgery. The principle of the surgery is to reduce the volume capacity of the spinal CSF reservoir and thus to decrease the compliance of the spinal compartment. Consequently, a smaller volume of CSF is shifted from the cranial to the spinal compartment when assuming an upright position. Schievink et al. demonstrated a positive effect of lumbar dural reduction surgery in a patient with intractable SIH but no evidence for CSF leakage (39). This is an invasive method, however, which should be reserved as a last option for selected patients.

## Conclusion

We propose a pathophysiological concept for the subgroup of SIH patients with typical cranial imaging findings and no evidence of CSF leakage after repeated and meticulous work-up. Pathologically increased spinal compliance as the main factor, in combination with predisposing factors such as decreased total and intracranial CSF volume, low CSF outflow resistance, and decreased venous pressure might cause a pathological orthostatic CSF shift resulting in intracranial hypotension and brain sagging even in the absence of CSF leakage. The compliance of the spinal compartment seems to be the most attractive therapeutic target.

## Data Availability Statement

The original contributions presented in the study are included in the article, further inquiries can be directed to the corresponding author/s.

## Author Contributions

JGo, LH, JB, and AR: design and conceptualized study and drafted manuscript. CJ, IZ, EP, JGr, and TD: revised manuscript for intellectual content. All authors contributed to the article and approved the submitted version.

## Conflict of Interest

The authors declare that the research was conducted in the absence of any commercial or financial relationships that could be construed as a potential conflict of interest.

## Publisher's Note

All claims expressed in this article are solely those of the authors and do not necessarily represent those of their affiliated organizations, or those of the publisher, the editors and the reviewers. Any product that may be evaluated in this article, or claim that may be made by its manufacturer, is not guaranteed or endorsed by the publisher.
